# Regulation of Toll-Like Receptor (TLR) Signaling Pathway by Polyphenols in the Treatment of Age-Linked Neurodegenerative Diseases: Focus on TLR4 Signaling

**DOI:** 10.3389/fimmu.2019.01000

**Published:** 2019-05-10

**Authors:** Shofiul Azam, Md. Jakaria, In-Su Kim, Joonsoo Kim, Md. Ezazul Haque, Dong-Kug Choi

**Affiliations:** ^1^Department of Applied Life Science & Integrated Bioscience, Graduate School, Konkuk University, Chungju-si, South Korea; ^2^Department of Integrated Bioscience & Biotechnology, Research Institute of Inflammatory Disease (RID), College of Biomedical and Health Science, Konkuk University, Chungju-si, South Korea

**Keywords:** polyphenols, MyD88, Toll-like receptor, NF-κB, neurodegenerative disease, inflammasome

## Abstract

Neuronal dysfunction initiates several intracellular signaling cascades to release different proinflammatory cytokines and chemokines, as well as various reactive oxygen species. In addition to neurons, microglia, and astrocytes are also affected by this signaling cascade. This release can either be helpful, neutral or detrimental for cell survival. Toll-like receptors (TLRs) activate and signal their downstream pathway to activate NF-κB and pro-IL-1β, both of which are responsible for neuroinflammation and linked to the pathogenesis of different age-related neurological conditions. However, herein, recent aspects of polyphenols in the treatment of neurodegenerative diseases are assessed, with a focus on TLR regulation by polyphenols. Different polyphenol classes, including flavonoids, phenolic acids, phenolic alcohols, stilbenes, and lignans can potentially target TLR signaling in a distinct pathway. Further, some polyphenols can suppress overexpression of inflammatory mediators through TLR4/NF-κB/STAT signaling intervention, while others can reduce neuronal apoptosis via modulating the TLR4/MyD88/NF-κB-pathway in microglia/macrophages. Indeed, neurodegeneration etiology is complex and yet to be completely understood, it may be that targeting TLRs could reveal a number of molecular and pharmacological aspects related to neurodegenerative diseases. Thus, activating TLR signaling modulation via natural resources could provide new therapeutic potentiality in the treatment of neurodegeneration.

## Introduction

Polyphenols are secondary metabolites of plants and serve to protect against a variety of pathogens, as well as ultraviolet damage. This phytochemical class of compounds also has a potential role in different oxidative stress-induced complications, such as cardiovascular disease, cancer and neurodegenerative diseases (1). Thus, a regular diet comprising frequent intake of polyphenol derivatives has been found to lower the risk of deposition of low-density lipoprotein (LDL), preventing endothelial coagulation and hindering atherosclerosis (2–5). Polyphenols are available in different kinds of fruits, vegetables or herbs and act as micronutrients. Approximately 8,000 or more members of this phytochemical group have been identified, and they originate from either phenylalanine or shikimic acid with a common phenolic group in their structural ring (6). Primarily, their classification includes phenolic acid, flavonoids, stilbenes and lignans (6).

However, aging and age-linked neurological complications are frequently observed and reaching epidemic levels due to day-by-day environmental or lifestyle modifications. At >60 years of age, different regions of the brain progressively and slowly lose cells due to the overexpression of cytokines, chemokines and neurotoxicity. This pathologic condition is featured by neurodegenerative diseases, such as Alzheimer's diseases (AD), Parkinson's disease (PD), multiple sclerosis (MS), Huntington's disease (HD), and amyotrophic lateral sclerosis (ALS) (7, 8). Several etiologies of such neurodegeneration are commonly associated with oxidative stress, neuroinflammation, mitochondrial dysfunction, protein aggregations and apoptotic factor activations (7). As such, researchers have attempted to understand the associated pathogenesis in this regard and to develop treatments; however, current approaches are not particularly promising and only symptomatic because in most neurodegenerative diseases, symptoms appear later. Thus, early preventive measures can interfere with disease progression and decrease suffering. One promising preventive attempt may be the inclusion of polyphenols in the regular diet, an approach that can reduce oxidative stress. The phenolic group of polyphenols interrupt the incessant oxidation in the cell by accepting an electron and forming a stable phenoxyl structure that breaks the formation of reactive oxygen species (ROS) (9). Thus, this group increases plasma antioxidant capacity, consequently reducing lymphocytic DNA damage, protecting cell components from degeneration (6, 10) and reducing the risk of oxidative stress-induced degenerative disorders. Moreover, polyphenols stimulate the Nrf2/ARE signaling pathway to enhance endogenous antioxidant component synthesis. This class of compounds also has the potential to modulate NF-κB-promoted neuroprotective activity (11).

Microglial cells and astrocytes are the primary sources of ROS. Microglial activation triggers neurodegeneration by activating and hypersecreting excitotoxic neurotransmitters that reduce ATP and growth factors in injured neurons (12). In that case, a potential anti-oxidant, such as polyphenols, may provide neuroprotection by inhibiting ROS generation and reducing auto-inflammatory responses. Therefore, polyphenols can act as both anti- and pro-oxidants, depending on their highly specific structure and cellular redox context, which may include either increased oxidant scavenging proteins or reduced oxidized proteins. For example, EGCG (Epigallocatechin gallate) improves mitochondrial function via antioxidative action (13). Besides polyphenols' ROS-scavenging ability, metal chelation and enzyme regulation also forms part of the mechanism of antioxidative action (14). Additionally, polyphenols can modulate the important pathogenesis of ND with its pleiotropic activity, including antioxidant properties. For example, polyphenols can modulate the NF-κB-mediated pathway to provide neuroprotection. In addition, polyphenols attenuate cognitive impairment, Aβ-aggregation and pro-inflammatory cytokines (15). While the actions of cytokines are well-known, including their inhibition exerting neuroprotection, in some cases, inhibition may exacerbate neuronal damage (16–18). Cytokine response in the CNS requires activation through a specific motion, while TLRs, as a part of the innate immune system, also regulate cytokine responses in the CNS. Therefore, this review aims to provide insight into natural compound-based TLR signaling intervention toward inflammatory cytokine overexpression, a process that may impact future neurodegeneration therapy.

### Polyphenols: Overview on Bioavailability and Permeability Through BBB

Naturally occurring polyphenols include four major classes: flavonoids, phenolic acid, stilbenes and lignans, with each member being further divided into different subgroups. Among these compounds, the flavonoids are the most comprehensive group, with a structural backbone of C_6_-C_3_-C_6_ and that contain an oxygenated heterocycle (19). Flavonoids are further sub-divided into 14 groups, including flavones, dihydroflavones, isoflavones and anthocyanidines (20). However, the pharmacological activity of different polyphenols depends on their affinity toward a complex formation with other groups, such as alcohols, acids or sugar, as well as their bioavailability (21).

The bioavailability of polyphenols widely differs from person to person due to the glycosylation pattern and degree of polymerisation. Because natural polyphenols often exist as esters, polymers or glycosylated forms, they need to go through hydrolyzation for absorption. In that case, gut microflora would help by the deglycosylation, dehydroxylation, and demethylation of polyphenols (22). For example, flavonoids are the most poorly absorbed glycosides that require deglycosylation in the small intestine by β-glucosidases enzymes to convert into aglycones and then be absorbed. The availability of aglycones in the circulation also differs due to the Phase I and II metabolism of oxidized and conjugated flavonoids (22, 23).

Absorption and bioavailability of polyphenols is also affected by biotransformation. For example, curcumin, after ingestion by mice, was detected in plasma within 15 min as dihydrocurcumin. However, at 1 h, it peaks as tetrahydrocurcumin and at 6 h, curcumin decreases as monoglucuronide (24). Another study detected trace amounts of curcumin and its metabolites in the circulation and organs of healthy humans, which showed a low impact on the modulation of chemotherapy-induced apoptosis (25). On the other hand, resveratrol transformed into glucuronoids and sulfates within 15 min of oral consumption and circulated for more than 9 h with a bioavailability of 1% following metabolism (26). Further, other dietary components, such as carbohydrate, protein, fats, and alcohols also affect absorption and the bioavailability of polyphenols. Fats in the diet enhance polyphenol absorption, while serum albumin potentiates cellular uptake and delays elimination.

Due to poor absorptivity, rapid metabolism and elimination, polyphenols have highly selective permeability across the blood-brain barrier (BBB) that limits their bioavailability in the CNS as well as their therapeutic efficacy. Although polyphenols can alter brain function through improving cerebral blood flow (27), changing multidrug-resistant protein-dependent influx and efflux mechanisms (28, 29) and direct modification of neuronal and glial activities, to exert these activities, they must also move inside the CNS and at an effective concentration. The BBB, in that case, is the critical regulator, which controls the entry and retention of nutraceuticals in the brain. There are several transport systems at the BBB, and some are particularly specific to allow nutrients, such as amino acids, glucose, vitamins and iron, for both influx and efflux into the brain. The same principle also applies for polyphenols to enter into the brain. However, due to their variability in stereochemistry and interaction affinity with efflux transporters, such as P-glycoprotein (PGP) at the BBB, their availability in the brain also differs (30). One flavonoid—naringin—has been detected at an effective concentration in the rat brain when co-administered with PGP inhibitors, but on peripheral administration it was undetected (31).

Permeability through the BBB may also vary due to the degree of lipophilicity. In that case, less polar polyphenols or their metabolites have increased permeability into the brain compared to more polar ones (32). For instance, quercetin-3-O-glucuronide, a red wine metabolite, was detected at substantial levels in the Tg2576 AD mice brain after chronic oral administration. That resulted in a significant decrease in Aβ generation and toxicity, consequently improving hippocampus-associated synaptic deficits (33).

The form of administration is also crucial to improve polyphenol bioavailability. Co-administration of α-tocopherol with EGCG, quercetin and rutin in the diet synergizes quercetin transport through the BBB but not the EGCG. Curcumin may provide a particularly suitable example for understanding the limitations to achieve therapeutic potential *in vivo* because its bioavailability is insufficient; thus, several delivery systems, such as nanoparticles, liposomes and micelles failed to improve its bioavailability (34). Hence, co-administration with piperine increased curcumin concentrations in the brain at 48 h compared to the kidney (5.87 vs. 1.16 mg) (35). On the other hand, oxyresveratrol improved protection against 6-OHDA better than resveratrol because it is BBB permeable and water soluble (36). Similarly, bioavailability of EGCG has been improved by using it in a pro-drug form [fully acetylated EGCG (pEGCG)], as well as when tested on 6-OHDA induced SH-SY5Y neuroblastoma cells. The results demonstrated an improved protection by pEGCG more than EGCG, most likely due to the activation of the Akt pathway and reduced caspase-3 activity (37). As such, improvisation in administration strategy would improve the pharmacotherapeutic potentiality of polyphenols for neurodegeneration.

### Polyphenols: Signaling Interference for Neuroprotection

The most common pathological feature of AD progression is Aβ-aggregation. Several reports suggest that different polyphenols are involved in the amelioration of AD by reducing Aβ-plaques. For example, some *in vivo* studies report that tea polyphenol can inhibit acetylcholinesterase as well as Aβ-aggregation (38, 39). Similarly, polyphenols extracted from grape seeds significantly attenuated oligomerized Aβ-peptide and neutralized tau protein folding to recover from cognitive dysfunction, both *in vitro* and *in vivo* (40–45). In a transgenic mouse model, tannic acid reduced Aβ-deposition via lowering β-carboxyl terminal amyloid precursor protein cleavage and controlling neuronal inflammation (46), while 7, 8-dihydroxyflavone activates TR-KB (tyrosine receptor kinase B) and reduces β-secretase enzyme during Aβ-synthesis (47), thus demonstrating recover memory in an AD model. However, a study of rutin on SH-SY5Y neuroblastoma cells revealed a substantial decline in oxidative stress, glutathione disulfide formation and cytokines, such as TNF-α and IL-1β (48). Luteolin also showed a similar effect by attenuating microglial activation in an LPS-induced primary neuron-glia study (Table 1) (51).

**Table 1:** Effect of different polyphenols in various neurodegenerative models (49).

**Different type and dose of polyphenols**	**Dose and mode of administration**	**Model used**	**Results obtained**	**References**
Apigenin	10 μM and 20 mg/kg; oral gavage	BV-2 microglial cell and ischemic mice	Suppressing p38 mitogen-activated protein kinase (MAPK), c-Jun N-terminal kinase (JNK) phosphorylation	(50)
Luteolin	5 μM	LPS-induced primary neuron-glia	Attenuated microglial activation and overproduction of TNF-α, NO and superoxide	(51)
Kaemferol	30 μM	Rotenone-induced SH-SY5Y cell and primary neuron	Enhanced mitochondrial output by autophagy	(52)
Myricetin	10^−9^ mol/L	MPP^+^-treated MES23.5 cells	Attenuate cell loss, intracellular ROS, and phosphorylation of MAPK kinase 4 and JNK	(53)
Quercetin	25–75 mg/kg; i.p	Rotenone-induced rats	Reducing dopaminergic cell loss in striatum	(54)
Catechin	10–30 mg/kg; i.p	6-OHDA-lesioned rats	Improved locomotor activity and rotational behavior, and increased dopamine content	(55)
Naringenin	80 μM and 70 mg/kg; oral gavage	6-OHDA-induced SH-SY5Y cell and mice	Increased Nrf2 protein and protect nigrostriatal dopaminergic neuron in neurodegeneration	(56)
Theaflavin	10 mg/kg; oral gavage	MPTP-induced mice	Reducing oxidative stress and improving motor function and dopaminergic expression in striatum and substantia nigra	(57)
Silymarin	1–10 μg/kg; i.v.	CI/Required-induced rat, stroke model	Ameliorate oxidative and nitrosative stresses and inflammation-mediated tissue injury impeding activation of proinflammatory transcription factors NF-κB and STAT-1	(58)
Juglanin	10–30 mg/kg; i.p.	LPS-induced C57B/L6 PD mice	Betterment of neuroinflammation-related memory impairment via interfering with TLR4/NF-κB signaling	(59)
Rutin	2–20 μM	AD model using SH-SY5Y neuroblastoma cells	Modulates production of proinflammatory cytokines by decreasing TNF-α and IL-1β	(48)
7, 8-dihydroxyflavone	5 mg/kg; i.p.	5XFAD mice of AD model	TrkB activation and improved AD-associated memory deficits; reductions in BACE1 expression and Aβ-aggregation	(47)
Xanthohumol	0.2 and 0.4 mg/kg; i.p.	MCAO-induced ischemic rats	Inhibits inflammatory responses via HIF-1α, iNOS expression reduction, and reduced apoptosis through impeding TNF-α, active caspase-3	(60)
Fisetin	50 mg/kg; i.p.	MCAO-induced ischemic mice	Protected brain tissue against ischemic reperfusion injury; inhibited infiltration of macrophages and dendritic cells into ischemic hemisphere; suppressed TNFα production	(61)

*CI/R, cerebral ischemic/reperfusion; MCAO, middle cerebral artery occlusion*.

In a study using SH-SY5Y cells, oxyresveratrol (36) enhanced the SIRT1 (silent mating type information regulation 2 homolog 1) gene and downregulated caspase-3, JNK and JTF (c-Jun transcription factors) to reduce neuronal damage. Similar neuroprotective action was demonstrated using ferulic acid via JNK pathway downregulation in an ischemia/reperfusion-induced mice model (62). In contrast, quercetin protects neurons by stimulating glutathione peroxidase (GPx), superoxide dismutase (SOD), Na (+), and K (+) -ATPase (62) and suppresses apoptosis in an *in vitro* PD model. Furthermore, it also reduced dopaminergic cell loss in rat striatum (Table 1) (54). Other polyphenols, such as baicalein, kaempferol, caffeic acid, and EGCG (52, 63–65) also revealed neuroprotective action in PD, both *in vitro* and in an animal model study. For example, mulberry fruit extracts modulated Bcl-2, caspase-3 and Bax, and showed an anti-apoptotic effect in an experiment on SH-SY5Y cells (66). Resveratrol was reported to have significant therapeutic value to activate SIRT1 in brown adipose tissue in a study on an N171-82Q transgenic mouse model for HD (63). Also, using an encephalomyelitis mouse model, resveratrol was found to inhibit neural loss without inducing immunosuppression (67). Juglanin, a flavonol derivative, in LPS-induced C57B/L6 mice potentially modulated IL-1β and TNF-α, and ameliorated neuroinflammation-related memory impairment, and neurodegeneration through impeding TLR4/NF-κB (59).

Dietary polyphenols modulate the NF-κB inflammatory pathway and attenuate Aβ-toxicity. Different flavonoids, such as quercetin, apigenin, and luteolin have been reported to suppress the NF-κB-pathway and result in inhibition of Aβ (68). Moreover, the isoflavone extracted from soybean reduced memory impairment in a neurodegenerative rat model via blocking NF-κB expression (69), while resveratrol and baicalin attenuated Aβ-induced neuronal inflammation through downregulating NF-κB signaling (70, 71). Thus, NF-κB is important not only in inflammation, but also for cell death events in cerebral ischemic injury. Silymarin, a flavonoid derivative, has been shown to protect against cerebral ischemia by inhibiting NF-κB and STAT-1 (signal transducer and activating transcription-1) activation in cerebral ischemic/reperfusion-induced rats, in a dose-dependent manner (1–10 μg/kg, i.v.) (58, 72). Apigenin also provided a significant neuroprotective effect in an ischemic mice model via suppressing JNK phosphorylation (50), whereas 20 mg/kg of apigenin reduced cerebral infarct volume significantly (Table 1).

Similarly, 2,3,4′,5-tetrahydroxystilbene-2-O-β-D-glucoside (TSG) of *Polygonum multiflorum* provides neuroprotection in cerebral ischemia by inhibiting NF-κB-signaling and activating SIRT1 (41, 73). Quercetin also inhibits NF-κB to protect the brain from oxidative stress or hypoxic damage (74), and a similar effect was demonstrated by catechin hydrate, baicalin, and fisetin (Table 1). Moreover, these phytochemicals were also found to inhibit IL-1β and TNF-α proinflammatory cytokine expression (61, 75, 76). Catechin also improved locomotion and increased dopamine in a 6-OHDA-lesioned rat (55). Continual investigation of polyphenols confirms their role as immunomodulatory agents because they can control inflammatory stimuli via downregulating NF-κB expression (46).

However, resveratrol demonstrated increasing Nrf2 (nuclear factor-2) expression. The Nrf2-pathway is involved in p53 gene expression, which leads to antioxidant protein encoding (46, 77). Further, resveratrol increases HO-1 (heme oxygenase-1) expression and downregulates the caspase-3 apoptotic enzyme (78). Similarly, protective action was also revealed by epicatechin in stroke and oxidative stress via upregulating Nrf2 (79). Additionally, a prenylated chalcone, xanthohumol, inhibits the HIF-1 (hypoxia-inducible factors-1) pathway, leading to neuroprotection (Table 1) (60). In a 6-OHDA-induced SH-SY5Y cell study, naringenin increased Nrf2 to protect dopaminergic neurons, while also providing the same effect in a neurodegenerative mice model as well (56).

### Toll-Like Receptors: Signaling and Expression in CNS

Toll-like receptors (TLRs) were first identified in the protein content in Drosophila. Later, their importance in providing innate immunity against microbial infection was recognized (80), and within the family, TLR4 is the first identified mammalian homolog. Unlike adaptive immunity, innate immunity is the first line of defense against anonymous pathogenic invasion, relying on molecular determinant sensing of, for example, pathogen-associated molecular patterns (PAMPs) (81–84). TLRs are a member of the pattern recognition receptor (PRR) group, a large group that includes both intracellular and extracellular receptor families, and sense PAMPs or DAMPs (damage-associated molecular patterns). TLR members are mostly expressed in microglia rather than astrocytes and neurons. However, in certain conditions, some members are expressed in astrocytes and a few in neurons, such as viral- or LPS-induced N9 microglia expressing TLR2 and differentiating astrocytes expressing TLR7 (85). Likewise, TLR4, although expressed in microglia often, are also produced in astrocytes and neurons in response to bacterial LPS (Table 2) (87, 88).

**Table 2:** Expression of different Toll-like receptors in the nervous system.

**Toll-like receptors**	**Microglia**	**Astrocyte**	**Neuron**
TLR1	+	–	–
TLR2	+	+	–
TLR3	+	–	+
TLR4	+	+	+
TLR5	+	–	–
TLR6	+	–	–
TLR7	+	–	+
TLR8	+	–	+
TLR9	+	+	+

“*+,” expressed; “–,” expression not detected (86)*.

A recent study suggests that increased TLR expression in the neuron can be or is probably linked with different physiological and pathological conditions. Analysis of a teratoma-forming cell line NT-2 (Human NTera2) found mRNA expression for TLR1, 2, 3, and 4; mRNA expression of TLR1-9 and protein expression for 2-4 from rat primary neuronal cells was also evident (89–91). Additionally, an *in vivo* study on murine mice showed mRNA expression of TLR1-8 (92) and the neuronal expression of TLR2 and 6, as well as in pathogenic conditions, such as parasitic infection, TLR2, 4 and 6–8 were expressed (92). Some researchers have found that both human and rat inflammatory neurons co-express TLR4 and CD14, a result which may be due to LPS action through TLR4/CD14 complex formation (93). However, TLR3 can be expressed in both central and peripheral neurons (94).

**Figure 1:**
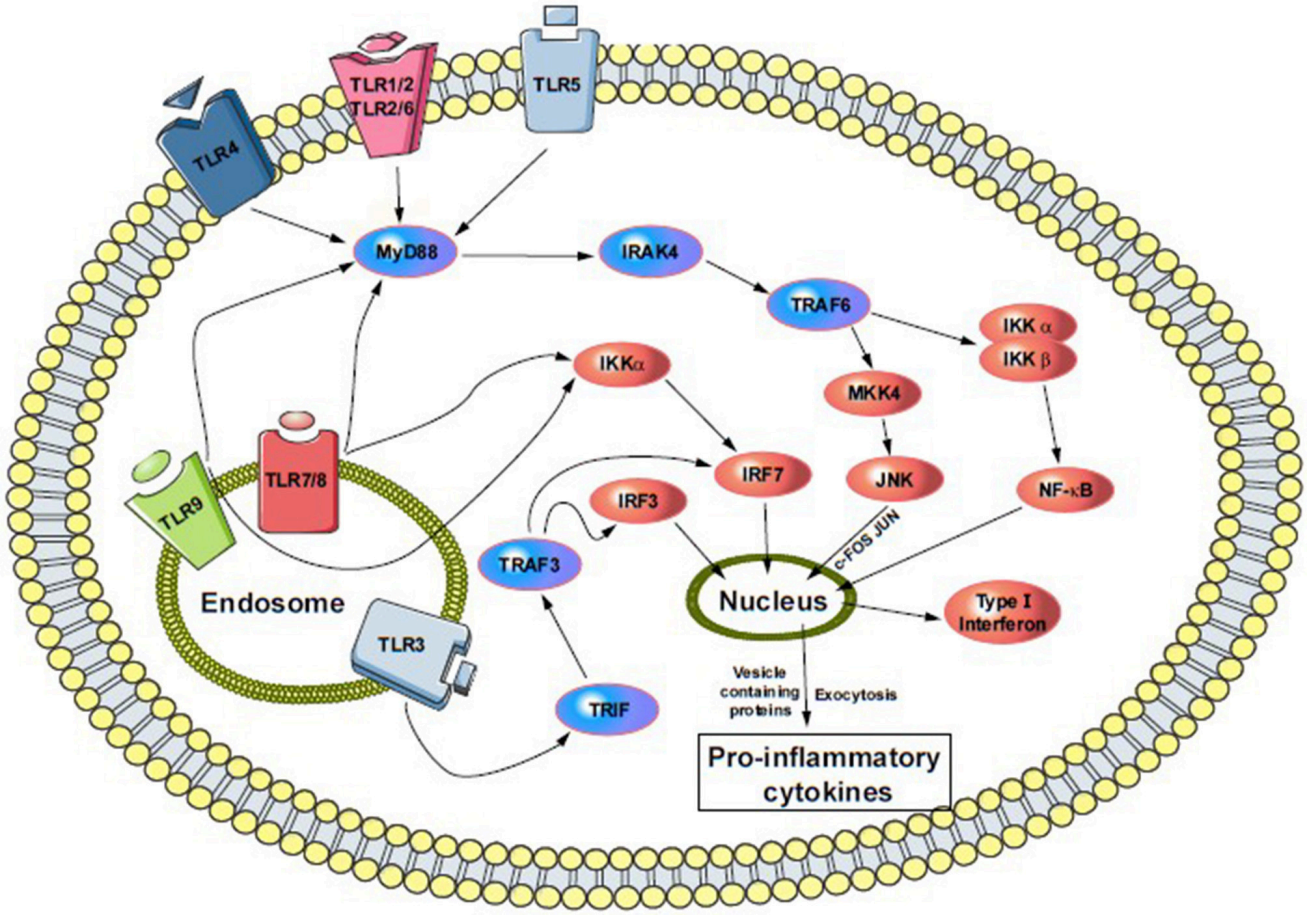
Cell surface and endosomal signaling pathway of TLRs. TLR4, TLR5, and heterodimers TLR1/2 and TLR2/6 sense bacterial invasion and initiate intracellular TLR-signaling pathway. Following the activation, each of them recruits several adaptors in the cytoplasm and activate MyD88-downstream. That means the activation of IRAK4 and phosphoryl IRAK1 that then bind to TRAF6 (not shown). TRAF6 then recruit MKK4 and IKKα/β pathway, where MKK4 initiate JNK and activate c-FOS and JUN, and push into the nucleus. While IKKα/β activates NFκB and its pro-inflammatory subunits and moves into the nucleus, similarly, endosomal TLRs (TLR3, 7–9) triggers the NFκB and MAPK pathways via involving MyD88 and IRAK4. Additionally, TLR3, MyD88 independently, recruit TRIF-pathway leading to the phosphorylation and dimerization of IRF7. Both, surface and endosomal pathway ultimately result in a production of type I interferon and release of proinflammatory cytokines. MyD88, myeloid Differentiation primary response 88; IRAK4, interleukin-1 receptor-associated kinase-4; TRAF6, TNF receptor-associated factor-6; MKK4, mitogen-activated protein kinase kinase-4; IKKα/β, IκK kinase; JNK, c-Jun N-terminal kinase; TIRF, TIR-domain-containing adapter-inducing interferon-β; IRF3/7, interferon regulatory factor-3/7.

TLR signaling is complex and depends on other protein and co-receptor pathway activation. Most members depend on the MyD88 (myeloid differentiation factor 88) pathway, except for TLR3 and TLR4. Both of them are unique in their functionality to activate IRF3 (interferon regulatory factor 3). For example, TLR4 activation through the MyD88-independent pathway also activates and recruits TRIF (TIR-domain-containing adaptor-inducing interferon-γ) and TRAM (TRIF-related adaptor molecule). Further, the signal cascade activates NF-κB and IRF3, and initiates IFN (type-I interferon) production. TLR3 activates through a TRIF-dependent pathway that recruits IKKs (IκB kinase), TBK1, and IKKε to begin activation of IRF3, and releases type-I IFN into vesicles (Figure 1) (91, 95). This pathway also activates IRF2 via phosphatidylinositol 3-kinase and AKT (91, 96). Other members, such as TLR7, 8 and 9, can also activate type-I IFN through a MyD88-dependent pathway (Figure 1).

Different descriptions in the above figure indicate that TLR2 and 4 affect neuronal differentiation and both are expressed in adult neural stem cells (97). Indeed, TLR4's absence enhances proliferation and neuronal differentiation, while the lack of TLR2 damages hippocampal neurogenesis (98). Both TLR2 and 4 modulate the cell fate of neuronal progenitors (91) via MyD88 and NF-κB signaling (Figure 1). However, NF-κB-dependent TLR signaling in neuronal cells is highly specific and their signaling in differentiated neurons has yet to be determined.

Furthermore, with respect to TLRs along with NOD-like receptor (NLRs) signals for inflammasome activation, both are almost identical in their structure and have similarities in the component and signaling pathways. However, following inflammasome activation, caspase-1 signaling cascade also becomes involved and mature IL-1β is released into extracellular vesicles. TLR activation by various ligands also leads to the recruitment of downstream pathway signaling via the MyD88 adaptor and activates NF-κB, which expresses the 31-kDa inactive precursor pro-IL-1β, in the cytosol. Meanwhile, inflammasome activates caspase-1 as an inactive 45-kDa zymogen, which is later catalyzed and activates. Thus, this compound comprises p20 and p10 subunits, both of which are assembled into a heterotetramer. The active caspase-1 cleaves pro-IL-1β and transforms into a 17-kDa biologically active IL-1β. Similarly, caspase-1 also cleaves pro-IL18, which unlike pro-IL-1β, is constitutively expressed (99, 100).

### TLRs: Intricate Role in Neurodegenerative Diseases

A number of studies on inflammatory markers have demonstrated the involvement of TLRs in aging-related neurodegenerative disorders, such as AD, ischemic strokes and multiple sclerosis. With age, the brain's pro-inflammatory gene transcription upregulates; therefore, TLR transcription levels change and participate in age-linked neurodegeneration. Moreover, they are also involved in brain trauma following injury, where glial cells activate and express different cytokines and chemokines near the injury area. In a mouse model of brain injury, TLR2 was found upregulated by microglia in the hippocampus zone. In contrast, TLR2 deficits reduce microglial activation, cytokine and chemokine expression (101, 102).

**Figure 2:**
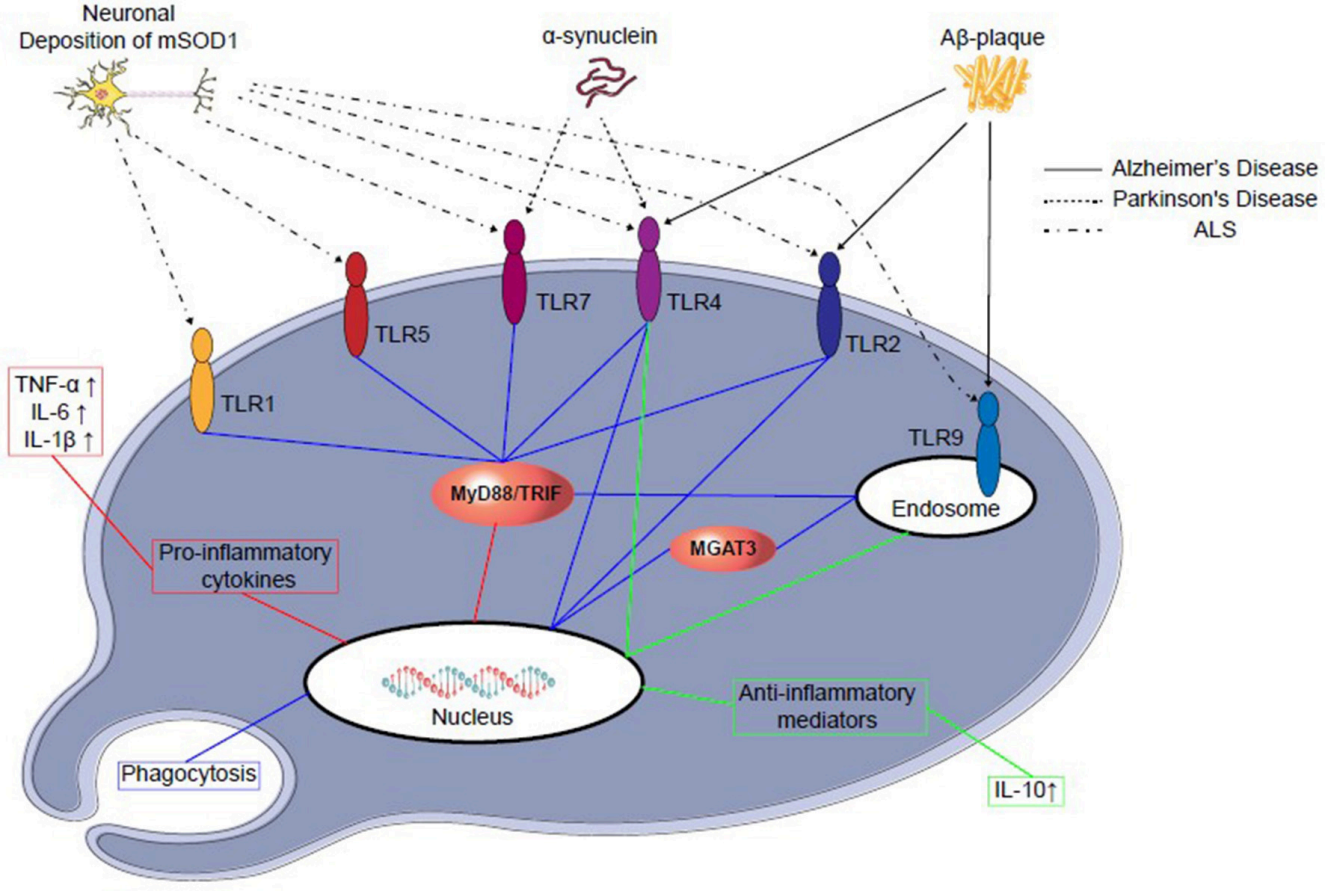
TLRs-signaling in microglial cells in different neurodegenerative disease progression. Abnormal amyloid deposition in different neurodegenerative diseases may activate microglial cells through TLRs. Microglial activation may lead to further neuronal damage through secretion of proinflammatory cytokines (red), such as IL-6 and TNF-α, or neuroprotection by secretion of anti-inflammatory cytokines (green), such as IL-10, which may prevent further neuronal death. Furthermore, recent reports suggest TLRs 2, 4, and 9 signaling may modulate the phagocytosis (blue) and clear the neurotoxic amyloid deposition (108). Aβ stimulation, mononuclear cells of normal subjects up-regulate the transcription of β-1,4-mannosyl-glycoprotein 4-β-Nacetylglucosaminyltransferase (MGAT3).

TLR4 is also profoundly involved in the glial cell expression and activation of NF-κB, as well as initiation of inflammatory cytokines, such as TNF-α, IL-1β, and IL-6 production in the brain in different injured animal models (103–106). Both TLR2 and 4 signaling are involved in the activation of glial cells and other inflammatory cytokines and are responsible for inflammation in the injured brain (107). However, in glioma—a glial cell tumor—TLR9 is expressed significantly and was found to be beneficial in a clinical study (Figure 2) (109–111).

#### TLR Involvement in AD

The most common pathophysiology of AD, an age-related neurodegenerative disorder, is the deposition of Aβ-plaques in the hippocampal region of the brain. Several AD model studies have also discussed the involvement of TLRs. For example, a survey showed significant TLR4 expression in glial cells surrounded by Aβ-plaques (112–114), with TLR4 polymorphism being proposed to have a protective role in AD (113, 115). Although the effects of TLR4-knockout on behavior or disease progression are yet to be documented, microglia-mediated TLR4 may be less efficient in a TLR4-knockout model to clear Aβ-plaques, leading to the overproduction or aggregation of Aβ (116, 117). It is evident that mouse microglia aggregate Aβ via TLR4 and cause neuronal death (115); thus, microglia require TLR4 for LPS-induced Aβ uptake (112, 117). As well, neurons, with the help of TLR4, respond to Aβ and AD-linked peroxidation and result in apoptosis (115).

TLR2 deficiency, however, aggravates cognitive impairments in an AD mouse model. This effect may be reversed by TLR2-expressing bone marrow-derived cells that can stimulate microglial clearance of Aβ from the brain (118, 119). Therefore, TLR2 may respond as bone-marrow-derived immune cells to protect from Aβ-aggregation. Furthermore, TLR2, TLR4, or TLR9 activating ligands have been reported to increase the uptake of Aβ by a microglial cell line (117). Another *in vivo* study reports that TLR2 and 4 are also required to activate microglia-mediated Aβ-plaques (120). Additionally, exposure of microglia to the TLR9 ligand, CpG DNA, protects neurons against Aβ toxicity and reduces Aβ aggregation-mediated memory impairment in mice (119, 121). Collectively, data on multiple TLRs suggest their activation in the AD brain cells and the well-known role that they have. For example, microglial TLR2, 4 and 9 may counteract the disease process by enhancing Aβ clearance, while activation of TLR4 in neurons can aggravate the condition with initiating oxidative stress and Aβ toxicity. Due to increased knowledge gathered with respect to the role of neuronal TLR4 in AD, it is important to explore this receptor function further in the AD-induced animal model or human tissue/cell line. As such, we can differentiate glial-mediated TLR4 responses from neuronal responses, as well as its role in the association of disease-specific protein aggregation and neuroinflammation or apoptosis.

#### TLR Involvement in PD

The various views regarding etiology of PD suggest that misfolded α-synuclein activates microglial cells, leading to inflammation, oxidative stress and finally, neurodegeneration. The misfolded α-synuclein is released from neural cells or oligodendrocytes, also known as PAMPs or DAMPs, by microglial TLR2 that ultimately activates the downstream pathway of MyD88 and NF-κB, triggers TNF-α, IL-1β and increases selective TLR expression (122–124). In one study, TLR4 has been found to interact with α-synuclein along with its uptake, proinflammatory cytokine release and enhancing oxidative stress (125). An MPTP-induced PD mouse model analysis interpreted neuroprotection due to the genetic absence of TLR4, supporting the significant role of TLR4 in the generation and progression of PD (126). Interestingly, TLR4 absence protected from dopamine downregulation with an increase in dopamine transport activity and significantly reduced α-synuclein-positive neurons in an MPTP-induced PD model. In that study, the absence of TLR4 also modulated NF-κB, AP-1, and NLRP3 inflammasome pathways, thus reducing the development of PD-associated neuroinflammation (127). However, the role of TLR2 and 4 during the progress of PD is particularly convincing, although complicated. Their activation of microglia can trigger neurotoxicity, while in other cases, they might be necessary to clear misfolded α-synuclein and act as a neuroprotector (Figure 2 and Table 3) (115). Therefore, both of them could be a potential therapeutic target for PD.

**Table 3:** TLR expression in different neurodegenerative disorders and their documented role.

**Disease**	**TLRs expression**	**Animal model**	**Human model**	**References**
Alzheimer's disease	TLR2 ↑	Both beneficial and deleterious	Beneficial	(115, 120, 128)
	TLR4 ↑	Both beneficial and deleterious	N/A	
	TLR7 ↑	TLR7 knockout improved spatial learning	N/A	
	TLR9 ↑	Reduced Aβ-aggregation	N/A	
Parkinson's disease	TLR2 ↑	Deleterious	Deleterious	(115, 128)
	TLR4 ↑	Deleterious	Deleterious	
	TLR5 ↓	Cognitive impairment	N/A	
	TLR9 ↑	Dopaminergic neuronal loss	N/A	
Amyotrophic lateral sclerosis	TLR2 ↑	Degeneration of motor neuron	N/A	(128, 129)
	TLR4 ↑	Deficiency improves motor function	N/A	
	TLR9 ↑	Deleterious	N/A	
Stroke	TLR2 ↑	Both beneficial and deleterious	N/A	(91, 130)
	TLR4 ↑	Deleterious	Deleterious	

“*↑,” increased; “↓,” decreased; “N/A,” not available*.

#### TLRs Involved in Cerebral Ischemia/Stroke

The involvement and pathway of innate immunity in the generation of ischemic tissue has gained significant attention among neuro-researchers in various fields in recent years. According to them, microglial activation is the main reason behind inflammation following cerebral ischemia, and TLR members control this activation to a significant degree (131). Furthermore, TLR2 and TLR4 are the most common in this regard, as they are thought to liberate pro-inflammatory cytokines with respect to immune response; thus, exacerbate ischemic injury and subsequent neuronal damage result.

During a stroke, blood flow is eventually reduced and generates several conditions, such as ionic imbalance, acidosis and excitotoxicity (132) due to lack of oxygen and glucose. Sequentially, the damage of cellular constituents and release of DAMPS that activate specific TLRs occurs (133). In experimental animals as well as in stroke patients, it has been shown that HMGB1, a DAMP protein and also a ligand of TLR2 and TLR4, is increased in serum (134–136). Also, anti-HMGB1 antibody demonstrates a significant reduction in the aggravation of ischemic damage via attenuating cytoplasmic MCAO (middle cerebral artery occlusion) (134, 137, 138). However, following cell death, Prx (peroxiredoxin protein) is released into the extracellular compartment and acts as a DAMP. Moreover, it activates TLR2 and TLR4, leading to inflammation through cytokine overproduction. Likewise, administration of the Prx antibody just after experimentally induced stroke significantly reduces infarct volume, indicating that Prx also activates TLR signaling to intensify cerebral ischemic injury (139, 140). The majority of TLR-focused research has used either a rat or mouse model, and most of them target TLR2 and TLR4. One study demonstrated that TLR2 was markedly upregulated in the mouse cortex and TLR2 knockout mice showed increased infarct volume and mortality compared to wild-type mice (139). In a more recent study, deficiency of TLR2 was found to reduce ischemic volume at an early stage; however, the volume later increased significantly in comparison to wild-type mice, indicating that TLR2 deficiency in the brain can delay ischemic lesions (141).

Similarly, another study involving TLR4-deficient mice reported reduced damage compared to controls following ischemia (142), or permanent occlusion of the middle cerebral artery (143). Meanwhile, several clinical studies also noted the critical role of TLRs in a stroke patient, particularly the involvement of TLR4 polymorphism in terms of stroke prevalence (130, 144). Some research also found a significant rise in TLR2 and TLR4 on peripheral monocyte after stroke (145–147). Together, these studies indicate that TLR2 and TLR4 play a critical role in cerebral ischemia/reperfusion injury and that their activation leads to the exacerbation of brain damage. Along with TLR2 and TLR4, increased TLR7 and TLR8 also has been noticed in blood samples of deteriorating stroke patients, but no role has been reported for TLR3 or TLR9 in ischemic injury (148, 149).

#### TLRs Involved in Multiple Sclerosis (MS)

TLRs are always decisive for their involvement in different neurological diseases, and several pieces of evidence suggest their critical role in the pathogenesis of MS. TLRs have been found to be expressed in the glial cells of CNS of patients suffering from MS (150, 151). Moreover, TLR2 expression is upregulated in peripheral blood mononuclear cells (PBMCs) from MS patients, with PBMCs from RRMS (relapsing-remitting MS) being hypersensitive to TLR4 activation (152). Furthermore, different studies using MS knockout models have outlined the crucial role played by TLRs and their signaling proteins. For example, TLR2 (153), TLR9 (154), MyD88 (154–156) and IRF-3 (157) deficiency resulted in protective effects in neuroinflammatory models, while TLR4 (156), TLR2 (118), and TRIF (158) deficiency presented aggravating disease, indicating the complex role of TLRs in inflammatory development in MS. Recent data from an experiment by Mellanby *et al*. demonstrate that TLR4-induced activation of DC (dendritic cells) promotes the function of pathogenic T cells in EAE (experimental autoimmune encephalomyelitis) (159), a result that supports the complicated role of TLRs in EAE development.

#### TLRs Involved in ALS

Amyotrophic lateral sclerosis (ALS) is a devastating and chronic neurodegenerative disease, characterized by the selective upper and lower motor neuron loss, while about 20–25% of ALS cases are due to different mutations in the SOD1 gene (160). The aberrant oligomerisation of mutant SOD1 (mSOD1) proteins in beta-sheet form may be responsible for the pathogenesis and progression of ALS; it has also been demonstrated that mice lacking this gene do not develop the disease (160, 161). As well, mSOD1 has also been demonstrated in mice for an elevation of TLR1, 2, 7, and 9, and mSOD1 in microglia released more superoxide, nitrate and nitrite, resulting in severe neuronal death (Figure 2 and Table 3) (128). One study demonstrated that mSOD1 activates in microglia via a MyD88-dependent pathway, with some analyses documenting the significant effect of MyD88 in an ALS model (162). Although no significant difference is visible in the life-span of MyD88 knockout and normal mice, MyD88 knockout mice had increased activated microglia and motor neuron loss, indicative of a link between MyD88 deficiency and neurotoxicity (162). In contrast, a recent study demonstrated blocking TLR2 and 4 signaling, inhibiting microglial activation following extracellular mSOD1 administration (163). However, the chronic systemic administration of LPS aggravates disease progression and motor neuron degeneration with the elevation of TLR2 expression, suggesting a correlation between TLR2 expression and motor neuron degeneration (164). Thus, targeting TLR may attenuate neurotoxicity in ALS and potentially impact therapy; however, there is no clear evidence for a specific TLR that may mediate this effect. Therefore, the potential link between TLR signaling and neurotrophic factor secretion increment from glial cells could be a therapeutic approach in ALS.

### Polyphenol-Based TLR-Signaling Pathway Targeting: A Neurodegeneration Therapeutic Approach

**Figure 3:**
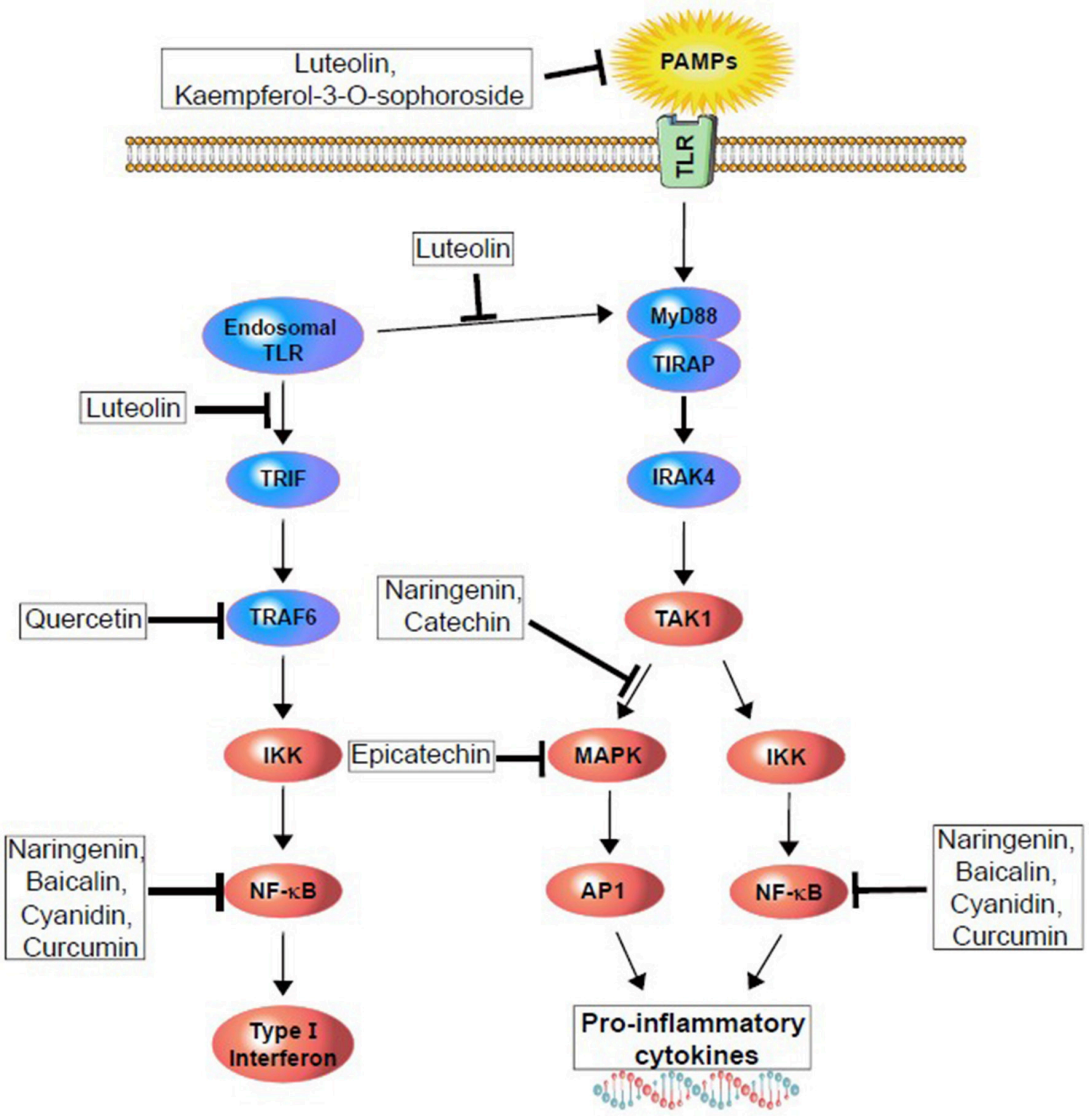
Polyphenols modulating upstream (TLR activation) and downstream (different kinase and transcription factors) pathway of surface and endogenous TLR to reduce or demolish pro-inflammatory cytokines and type I interferon generation.

Polyphenols are natural resources, potentially contributing to different therapeutic conditions with their anti-inflammatory and anti-oxidant properties, as well as interrupting the TLR4-signaling pathway. For example, green tea polyphenols have been examined to understand their effect on human periodontal inflammation induced by LPS at the pathogenic dose, with reported reduced TLR4 secretion and expression at both the mRNA and protein levels. That same extract was also reported to restore (150) standard hydrogen peroxide and hypochlorous acid, as well as to reduce the mRNA expression of TLR4 and IκK (165). Thus, polyphenols can decrease inflammation via TLR4 signaling pathway modulation (Figure 3 and Table 4).

**Table 4:** Different active polyphenols and their pharmacological attribution through TLR-signaling intervention.

**Active polyphenols**	**Polyphenols class**	**TLR intervention**	**Downstream signaling intervention**	**Response**	**References**
EGCG	Catechin type; belongs to the flavanols	TLR4 expression through 67LR	Inhibits MAPK and NF-κB	Inhibits LPS induced activation of downstream signaling and consequent inflammatory responses	(166)
Resveratrol	Stilbenes	TLR4 ligand	Inhibit downstream phosphorylation of STAT1 and 3	Reduced macrophages and microglial activation	(71)
Kaempferol-3-O-sophoroside	Flavonoids	Cell surface TLR2 and 4	Inhibit HMGB1 induced proinflammatory responses	Inhibits HMGB1-mediated proinflammatory cytokine production	(167)
Quercetin	Flavonols (Flavonoids)	TLR/NF-κB signaling pathway	Reduced IL-6 production and NF-κBp65 nuclear translocation	Downregulates inflammatory enzyme production	(168)
Naringenin	Flavanones (Flavonoids)	TLR2 and 4	MAPK pathway	Downregulation of TNF-α, IL-1β, IL-6 and other co-related inflammatory cytokines	(169)
Curcumin	Curcuminoids	TLR4	MyD88 and NF-κB downstream signaling	Reduce activation of microglia/macrophages and neuronal apoptosis	(170)
Silymarin	Flavonoids	TLR4	Inhibit TNF-α, IL-6 and IL-1β production	Attenuate deterioration of the nigral degeneration during PD	(171)
Epicatechine	Flavanols	TLR4	Inhibits MAPK and NF-κB	Reduce neuronal apoptosis	(172)
Isoliquiritigenin	Isoflavonoids (Flavonoids)	TLR4	Inhibits IRF3 activation	Decrease inflammatory gene expression	(173)
Soybean Isoflavones	Isoflavones	TLR4	Inhibits NF-κB p65 expression in the brain tissue	Reduced Aβ (1–42), as well as cytokine cascade and inflammatory response and improved learning and memory	(69)
Luteolin	Flavones (Flavonoids)	TLR3 and 4	TBK1 kinase and IRF3 phosphorylation	Modulated TRIF-dependent inflammatory responses	(174)
Catechin	Flavanols (Flavonoids)	TLR2	Downregulates p38MAPK and NF-κB p65	Reduced pro-inflammatory mediators and phosphorylation of their signal transduction	(175)
Fisetin	Flavonoids	TLR4	Suppress NF-κB activation and JNK/JUN phosphorylation	Neuroprotection in cerebral ischemia	(61)
Baicalin	Flavonoids	TLR2 and 4	Reduce the expression of NF-κB and serum content of TNF-α and IL-1β	Neuroprotection in cerebral ischemia	(75)

Neuroinflammation leads to the progress of neurodegeneration. In this aspect, TLRs play an essential role in several CNS disorders, and different studies have reported that TLR4, among other TLRs, are a frequent contributor to neuronal death, blood-brain barrier damage, oedema and ischemic brain injury (143, 176). Thus, the TLR4/NF-κB-signaling pathway plays a vital role in the activation of a different inflammatory gene expressing cytokines, chemokines such as COX-2 and MMP-9, and causes cerebral inflammation, as well as leading to secondary brain injury following traumatic brain injury (176–179). This upregulation of different cytokines or chemokines could also activate microglia; consequently, inflammatory cells infiltrate into the brain and may cause neuronal loss (180, 181). Recently, TLR4 was found to play a role in ethanol-induced inflammatory signaling. The study demonstrated that a TLR4 knockdown model abolished both MAPK and NF-κB-pathways and inflammatory mediators produced by astrocytes (182, 183). Also, use of quercetin, loaded into nanoparticles, improved their passage through the BBB and prevented AD progression via attenuating the TLR4-involved pathway (184). It also reduced inflammatory cytokine production by inhibiting TLR2 and 4 expression (168). Therefore, targeting TLR4 may be a particularly useful and novel strategy to treat neurodegenerative disorders.

Resveratrol, as earlier mentioned, is a potential neuroprotective and anti-inflammatory polyphenol, and under observation for the treatment of AD, inhibits murine RAW 264.7 macrophages and microglial BV-2 cells targeted by TLR4 ligand. Additionally, resveratrol inhibits downstream phosphorylation of STAT1 and STAT3 stimulated by LPS (71). Park and Yoon reported that isoliquiritigenin, a flavonoid derivative, inhibits LPS-induced TLR4 dimerization in RAW 264.7 macrophage lines. Therefore, it inhibits NF-κB and IRF3 activation, as well as COX-2 and inducible NO synthase expression (173). Similarly, luteolin suppressed activation of IRF3 and NF-κB induced by TLR3 and TLR4 agonists via the TRIF-dependent pathway, resulting in decreased expression of TNF-α and IL-6 in macrophages (174). These results indicate that polyphenols have the ability to modulate the TLR-pathway through TRIF-dependent signaling and result in potential attenuation of inflammatory cytokines. In a recent study, it was reported that silymarin pre-treatment significantly reduced overexpression of TLR4 in SNc induced by 6-OHDA in a PD rat model (171).

Cur (Curcumin) is a polyphenolic compound that has been used as a cooking ingredient for centuries. It has been noted for its potential in terms of anti-viral, antioxidant, antidiabetic and anti-inflammatory roles (185–187), and also with respect to its potent suppression of the TLR4-MAPK/NF-κB pathway (Figure 3). In an *in vitro* study, Cur was found to suppress NF-κB-mediated pro-inflammatory stimulation (188) and also inhibited LPS-induced IRF3 activation via MyD88 and TRIF-dependent pathways. However, another study with TLR4 targeted mice showed that 100 mg/kg treatment of Cur significantly reduced TLR4-positive microglia/macrophages and other inflammatory mediators' release, which are responsible for neuronal apoptosis. These results indicate that post-injury administration of Cur decreases acute activation of microglia/macrophages and neuronal apoptosis through intervening in the TLR4/MyD88/NF-κB-signaling pathway (Table 4) (170, 187). Cur can cross the BBB and thus, provide pharmacological activity efficiently, as demonstrated by Yang et al. (189). A recent study showed that Cur attenuates homodimerization of TLR4, which is necessary to trigger downstream cascade pathways (190). Thus, Cur can reduce inflammatory damage through TLR4 pathway modulation, which has since been confirmed in experimental models of brain injury (191–193).

However, upon microbial invasion, MAPK signaling pathways are activated to produce inflammatory mediators via TLR response, in turn activating down-regulation of p38 and NF-κB. In a study conducted by Yilma et al. naringenin was shown to inhibit TLR2 and 4 signaling (169), resulting in attenuation of pathogen-induced neuroinflammation. Moreover, EGCG and epicatechin also inhibit MAPK and NF-κB activation by attenuating TLR4 signaling, whereas catechin TLR2 signaling downregulates MAPK and NF-κB activation (166, 172, 175). Therefore, it reduces pro-inflammatory mediator activation and phosphorylation, as well as consequent neurodegeneration. A recent study reports that epigallocatechin gallate (EGCG) treatment prevents neurological pain via suppressing TLR4 cascades in a neuropathic rat model (194). Moreover, EGCG is one of the potent flavonoids found in green tea and is reputed for its ability to provide neuroprotection (195, 196). In an LPS-induced neuroinflammation mouse model, neurogenesis significantly decreased neuronal stem cell differentiation and proliferation. Additionally, microglial cells accumulated to initiate the LR4/NF-κB-signaling pathway in the hippocampus of mice. EGCG treatment showed an overall beneficial effect in this study with neurogenesis by inhibiting the TLR4/NF-κB-signaling pathway (197).

TLRs are critical elements of the innate immune system, and recent studies demonstrated their involvement in different brain injury-derived neurodegeneration processes. However, neuroinflammation plays an important role and leads to the development of neurodegenerative diseases, such as AD, PD, or MS. Indeed, several inflammatory markers, such as chemokines, cytokines or proteins in acute phase are upregulated and lead to inflammation, and these markers also prevail in neurodegenerative diseases, including AD (198–200). Additionally, TLR4 signaling pathways are involved and control these markers' upregulation. Thus, targeting TLR4 may represent an important therapeutic strategy to prevent neurodegenerative disorders mediated by different inflammatory markers (18, 182).

## Concluding Remarks and Future Aspects

Neurodegeneration is a pathological condition that includes the activation of different neuronal inflammatory cytokines and chemokines cascade, release of endotoxin and autoimmune disturbances and the overproduction of mitochondrial ROS. Here, a separate context was discussed to correlate the significance of NF-κB in the CNS and its regulation through TLR members. Further, recent approaches using polyphenols in the treatment of neurodegeneration were also discussed. Several polyphenolic compounds have been found to show promise for attenuating neurodegenerative disorders via involving interrelated mechanisms. However, they more likely target TLR4-linked pathway modulation to reduce inflammatory progression. There is growing evidence for the involvement of TLR4 in the etiology of different neuropsychiatric diseases; however, the source of TLR4 activation is yet to be determined. There appears to be two major pathways involved in TLR4 activation: either Gram-negative gut Enterobacter translocation or excessive production and release of ROS due to anonymous infection. In contrast, there are insufficient data regarding TLR4 dependent or independent cytokine effects and polyphenols' role on them in the progression of neurodegenerative diseases, while abundant investigation has been made regarding the role of cytokines in the pathogenesis of the same disorders.

Although neurodegeneration is a growing threat, there are only a few clinically relevant therapeutics for ND available, and they are for symptomatic treatment only. In this position with pathological concern and limited treatments, alternative and preventive therapeutics are rational to control the occurrence and progression of NDs. Some of them are under clinical investigation for therapeutic efficacy in neuropathological conditions; however, many more are expected to be tested in clinical trials for their *in vitro* and *in vivo* roles. Indeed, neurodegenerative diseases are complicated cases and involve several signaling cascades, but the role of Aβ-plaque aggregation and production of inflammatory cytokines and chemokines is also essential. In this case, several polyphenols have been shown to significantly attenuate Aβ-plaques and inflammatory cytokine and chemokine production via intervening different signaling pathways, explicitly targeting the TLR4/NF-κB-signaling pathway in AD, PD, MS, or stroke. Engagement of TLR along with another innate immune member, the NLR family, is also an important factor to release cytokines and to form a multiprotein inflammatory complex, the inflammasome. This emerging view is also important with respect to host response to pathogenic stimuli, and mature IL-1β release is a suitable example of this process, which aggravates the neurodegeneration. Therefore, future work should also focus on this area to determine precise signaling pathways and mechanisms, leading to comprehension of disease phenotypes and searches for effective therapeutics.

Based on a number of recent investigations, it is clear that polyphenols are promising, and their approaches involve TLR4 modulation to control NDs. Polyphenols have been found to reduce mRNA expression of TLR4 and IκK, while enhancing the MyD88-dependent TLR4/NF-κB-signaling pathway. However, this article attempted to describe the involvement of TLR4 in neurodegeneration and the role played by polyphenols via intervening in this pathway. Indeed, while polyphenols' action against innate immunity may be beneficial, the innate immune response is necessary under different CNS pathological conditions, where TLR4 activation can be neuroprotective. Although TLR4 removes Aβ-plaques by microglia via controlling phagocytes, TLR4 cytotoxicity has also been found to be significant in several studies. Therefore, it is necessary to elucidate TLR4s' complex signaling in the brain to gain control over inflammation-induced NDs. Targeting TLR4 would provide a highly suitable treatment approach, with significant implications in the designing of novel therapeutics for these particular diseases.

## Author Contributions

SA, D-KC, MJ, and I-SK conceptualized and designed the study. D-KC also supervised and corresponded. SA reviewed the literature, wrote the manuscript and compiled tables. SA and I-SK drew the figures. MEH and JK helped in revising the paper. All authors read and approved the final manuscript.

### Conflict of Interest Statement

The authors declare that the research was conducted in the absence of any commercial or financial relationships that could be construed as a potential conflict of interest.

## Abbreviations

ND: neurodegenerative diseases
AD: Alzheimer disease
PD: Parkinson disease
MS: multiple sclerosis
HD: Huntington disease
ALS: amyotrophic lateral sclerosis
CD40L: cluster of differentiation 40 ligand
SIRT1: silent mating type information regulation 2 homolog 1
SN: substantia nigra
JTF: c-Jun transcription factors
GPx: glutathione peroxidase
GSK-3β: glycogen synthase kinase 3β
CREB: cAMP response element binding proteins
TSG: 2,3,4′,5-tetrahydroxystilbene-2-O-β-D-glucoside
Nrf2: nuclear factor-2
HMGB1: high mobility group box 1 protein
PAMPs: pathogen-associated molecular patterns
DAMPs: damage-associated molecular patterns
PRRs: pattern recognition receptors
MyD88: myeloid differentiation factor 88
IRF3: interferon regulatory factor 3
TRIF: TIR-domain-containing adaptor-inducing interferon-γ
TRAM: TRIF-related adaptor molecule
IFN: type-I interferon
IKKs: IκB kinase
NPC: neural precursor cells
EGCG: epigallocatechin gallate
IRAK4: interleukin-1 receptor-associated kinase-4
TRAF6: TNF receptor-associated factor-6
MKK4: mitogen-activated protein kinase
kinase-4 IKKα/β: IκK kinase α/β
JNK: c-Jun N-terminal kinase
IRF3/7: interferon regulatory factor-3/7.
